# Comparative analysis of COVID-19 critically ill patients across four pandemic waves in Greece

**DOI:** 10.2478/jccm-2025-0036

**Published:** 2025-10-31

**Authors:** Stelios Kokkoris, Aikaterini Goufa, Dimitrios Tsilivarakis, Fotios Kavallieratos, Georgia Minatsi, Despoina Papadaki, Aikaterini Pranti, Spyros Zakynthinos, Anastasia Kotanidou, Christina Routsi

**Affiliations:** National and Kapodistrian University of Athens, Medical School, First Department of Critical Care Medicine, Evangelismos Hospital, Athens, Greece; National and Kapodistrian University of Athens, First Department of Medicine, Laiko General Hospital, Athens, Greece

**Keywords:** COVID-19, pandemic waves, SARS-CoV-2 variants, ICU, age, older adults, mortality

## Abstract

**Introduction:**

There is limited information about trends in mortality of intensive care unit (ICU) patients with Coronavirus Disease-2019 (COVID-19) throughout the entire pandemic period.

**Aim:**

We compared the ICU mortality among the four consecutive waves of the pandemic, according to the virus variant predominance.

**Methods:**

This is a retrospective study of prospectively collected data extracted from our COVID-19 clinical database. All adult patients with confirmed SARS-CoV-2 infection, consecutively admitted to our ICU from March 2020 through April 2022, were included. For the analysis we used the dates of the four periods of the pandemic, according to the predominance of different SARS-CoV-2 variants in Greece. Kaplan-Meier and Cox proportional hazards analyses were used.

**Results:**

In total, 805 patients [median (IQR) age 67 (56–76) years, 68% males] were included. APACHE II, Charlson, and SOFA scores were 14 (11–19), 3 (2–5) and 7 (4–9), respectively; 674 (84%) patients required invasive mechanical ventilation. ICU length of stay was 15 (8–29) days, and mechanical ventilation duration was 11 (4–24) days. ICU and hospital mortality was 48% and 54%, respectively. Kaplan-Meier survival curves revealed no significant differences in ICU mortality among the four waves. Age, malignancy, chronic pulmonary disease and SOFA score were independent predictors of ICU mortality, but the pandemic waves themselves were not. Age had a significant impact on ICU mortality across all waves.

**Conclusion:**

The effect of COVID-19 wave (and consequently of the SARS- CoV-2 variant) on ICU mortality seems to be trivial, and therefore our focus should be shifted to other risk factors, such as age and comorbidities. These findings along with those of other studies could be useful for modelling the evolution of future outbreaks.

## Introduction

Novel coronavirus disease 2019 (COVID-19) pandemic caused by the severe acute respiratory syndrome coronavirus 2 (SARS-CoV-2) broke out in China in December 2019 and rapidly disseminated worldwide causing more than 7 million confirmed deaths so far [1, 2]. The major clinical complications in patients with COVID-19 are acute hypoxemic respiratory failure and multiple organ failure requiring intensive care unit (ICU) admission. Although COVID-19 is currently considered as endemic with periodic epidemics throughout the world, the disease still ranks as the 10^th^ top cause of death according to a recent Centers for Disease Control and Prevention (CDC) report [3].

The emergence of new SARS-CoV-2 variants contributed to the incidence of multiple pandemic waves worldwide. The most globally dominant variants included: the Alpha variant, first documented in September 2020, the Delta variant, first detected in December 2020, and the most recent Omicron variant, documented in November 2021 [4]. In Greece, since the first confirmed case of COVID-19 in late February 2020, the nation has experienced four distinct pandemic waves due to the emergence of different SARS-CoV-2 variants. By the end of 2022, these waves had resulted in a total of 34,779 recorded deaths nationwide [5, 6].

Advancements in pharmacological treatments for severe COVID-19 [7, 8] which have been progressively incorporated into clinical practice, as well as improvements in non-pharmacological strategies, such as the expanded use of high-flow nasal oxygen (HFNO) [9] and the adoption of prone positioning [10], could collectively account for a significant impact on patient outcomes over time. Furthermore, potential differences in the virulence of the predominant SARS-CoV-2 variant during each pandemic wave, together with the increasing prevalence of immunity - either through vaccination or prior infection - could have also influenced ICU outcomes in severe COVID-19 cases throughout the pandemic [11, 12].

Following the initial COVID-19 wave, several studies worldwide have explored changes in mortality between pandemic waves in patients admitted to the ICU due to COVID-19. However, the majority of these studies have predominantly focused on the first year of the pandemic [ 13, 14, 15, 16, 17, 18, 19, 20], whereas data about trends in mortality throughout the COVID-19 pandemic, including all subsequent waves, among critically ill patients is scarce [ 21, 22,23].

The evolution of mortality over time is controversial. While some studies have demonstrated a decline in mortality rates [13, 14], others have reported no significant changes over time [ 16, 20, 24]. Furthermore, older adults consistently exhibited higher mortality rates compared to younger individuals throughout the pandemic [25, 26].

The primary objective of the present study was to compare the characteristics and clinical outcomes of critically ill patients admitted to our ICU due to COVID-19, across the four consecutive pandemic waves of the disease in Greece, defined according to the SARSCoV-2 variant predominance. The secondary aim was to investigate the association of age with ICU mortality within each pandemic wave.

## Materials and Methods

### Study population

This is a retrospective study of prospectively collected data extracted from our COVID-19 clinical database, formed at the beginning of the pandemic in March 2020. All adult patients with confirmed SARS-CoV-2 infection, consecutively admitted to our ICU from March 2020 through April 2022, were included. This is a 38-bed university ICU, which had been expanded to 64 beds at that period, operated as a COVID-19 ICU, at a large tertiary care center of 1000 beds in Athens, Greece. SARS-CoV-2 infection was confirmed by a positive real-time reverse transcriptase-polymerase chain reaction (RT-PCR) test of upper or lower respiratory tract specimens. ICU admission criteria were as follows: Intubation due to COVID-19 respiratory failure, high flow nasal oxygen or non-invasive ventilation requirement for deteriorating respiratory failure, COVID-19 related circulatory shock. Patients who died within 24 hours post admission were excluded from the study. Our ICU did not face any difficulties in terms of resources availability or ICU beds shortages, due to the opening of additional ICU beds. As a result, all COVID-19 patients requiring ICU admission, regardless of age and accompanying diseases, were transferred to the ICU timely. The study encompassed four COVID waves, each corresponding to specific periods of SARS-CoV-2 variants, identified with RT-PCR-based nucleic acid amplification tests (NAATs).

*Definition of epidemic waves in our country:* For the analysis we used the dates of the four periods of the pandemic, according to the predominance (i.e., present in more than 50% of samples) of different SARS-CoV-2 variants in Greece, as it has been proposed by Malli et al [5]. In particular: wave 1, from March 2020 to January 6, 2021, where the original (wildtype) variant was prevalent; wave 2, from January 7, 2021 to July 4, 2021, when the Alpha variant (B.1.1.7 Lineage) was prevalent; wave 3, from July 5, 2021 to December 19, 2021, where the Delta variant (B.1.617.2 Lineage) was prevalent; and wave 4, from December 20, 2021 to the end of the examined period (April 2022) where the Omicron variant (B.1.1.529 Lineage) was prevalent.

### Data collection

We recorded demographics, blood chemistry tests, scores of illness severity, comorbidities, mechanical ventilation requirement, inotropic/vasoconstrictive agents use, vaccination status, requirement for continuous renal replacement therapy (CRRT), use of extracorporeal membrane oxygenation (ECMO), length of mechanical ventilation, as well as ICU length stay (LOS) and outcome. Routine blood tests, arterial blood gases and respiratory parameters in mechanically ventilated patients, concomitant medications including dexamethasone treatment added to the standard of care treatment from mid-July 2020, remdesivir or immune-modifying treatments were also recorded.

Acute Physiology and Chronic Health Evaluation (APACHE) II [27], Sequential Organ Failure Assessment (SOFA) [28], and Charlson Comorbidity Index [29] scoring systems were calculated on ICU admission. Comorbidities included hypertension, diabetes mellitus, obesity, cardiovascular disease, chronic pulmonary disease, chronic kidney disease and active malignancy. Shock was defined as follows: persisting hypotension (systolic blood pressure < 90 mmHg and/or mean arterial pressure < 65 mmHg), despite adequate volume resuscitation, necessitating use of vasoactive agents [30].

Patient management followed the guidelines previously reported [31]. ARDS treatment guidelines [32], including prone position ventilation and conservative fluid management, were followed. The study protocol was approved by the Ethics Committee of our hospital (Protocol Number 116/2021). Informed consent was not required for this study due to its retrospective and observational design, which relied on the collection of anonymized data.

### Statistical Analysis

Numerical variables are presented as median and interquartile range (IQR), and were analyzed using the Mann-Whitney U test or Kruskal-Wallis test. Categorical variables are expressed as n (%), and were analyzed by chi-squared test. We further stratified the population according to their age quartiles (18–56, 57–67, 68–76, 77–103 years, respectively). Kaplan-Meier survival analyses were performed to evaluate the ICU all-cause mortality of age quartiles or pandemic waves, respectively. Univariate and multivariate Cox proportional hazards models were used to calculate the hazard ratio (HR) and the 95% confidence interval (CI) for ICU outcome. The adjusted analysis included sex, SOFA score, pandemic waves, age quartiles, treatment (dexamethasone, remdesivir, tocilizumab), comorbidities (diabetes, hypertension, coronary artery disease, chronic kidney disease, neoplasm, COPD). Analyses were conducted with SPSS software (version 24). All P values are two-sided, and the level of significance was set at P < 0.05.

## Results

### Overall cohort

**Fig. 1.**
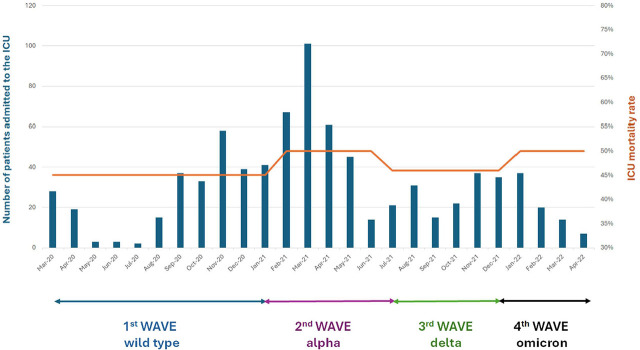
Distribution of ICU admissions, corresponding variant predominance across different waves, and mortality throughout the pandemic. Abbreviations: ICU, intensive care unit.

A total of 805 patients admitted to the ICU throughout the pandemic were included in the analysis. The first wave accounted for 243 (30%) admissions, the second for 300 (37%), the third for 158 (20%) and the fourth for 104 (13%), **Figure 1.** Sixty eight percent of patients were males, and their median (IQR) age was 67 (56–76) years. APACHE II, SOFA and Charlson scores were 14 (11–19), 3 (2–5) and 7 (4–9), respectively. In total, 674 (84%) patients required invasive mechanical ventilation during ICU stay; 588 (73%) patients were intubated already on admission; 238 (30%) of patients required CRRT. ECMO was used in eight cases. The ICU length of stay was 15 (8–29) days, whereas the duration of the mechanical ventilation was 11 (4–24) days. Dexamethasone was administered in 79%, remdesivir in 49% and tocilizumab in 5% of the patients. The overall ICU and hospital mortality rates were 48% and 54%, respectively.

### Comparisons among the four pandemic waves

Table 1 shows the demographics, comorbidities, treatments and outcomes distribution among the four pandemic waves. There were no significant differences in age, gender, and comorbidities among the four waves. For patients admitted to the ICU during the original SARS-CoV-2 strain, Alpha, Delta, and Omicron time periods, the ICU mortality rates were 45%, 50%, 46% and 50% respectively, p=0.645, Figure 1. Hospital mortality and CRRT need were significantly higher in the second and fourth waves in comparison to the other two waves. Specific treatments (dexamethasone, remdesivir and tocilizumab) were administered in significantly lower proportion of patients in the first wave compared to the other three waves. Use of mechanical ventilation and the presence of shock on ICU admission were higher in the fourth wave compared to each one of the other three waves. As expected, waves three and four had significantly higher vaccination rates compared to waves one and two.

**Table 1.** Demographics, comorbidities, treatments and outcomes distribution among the four pandemic waves

**Total n=805**	**1st wave n=243**		**2nd wave n=300**		**3rd wave n=158**		**4th wave n=104**		**P value**
Demographics									
Sex, male	176	(72)	200	(67)	100	(64)	71	(69)	0.28
Age, years, median (IQR)	67	(57–76)	68	(59–75)	65	5(0–76)	68	(56–76)	0.21

Severity scores									
Charlson index, median (IQR)	3	(2–5)	3	(2–5)	3	(1–5)	3	(2–5)	0.08
APACHE II score, median (IQR)	13	(9–19)	15	(12–19)	14	(11–19)	15	(12–18)	0.004
SOFA score, median (IQR)	6	(2–9)	7	(6–9)	7	(5–8)	7	(6–8)	0.002

Comorbidities									
Hypertension	101	(42)	131	(44)	58	(37)	40	(38)	0.50
Diabetes	65	(27)	82	(27)	33	(21)	24	(23)	0.42
Obesity	29	(12)	34	(11)	26	(16)	15	(14)	0.42
Cerebrovascular disease	65	(27)	71	(24)	34	(21)	32	(31)	0.32
COPD	25	(10)	51	(17)	18	(11)	12	(11)	0.10
Malignancy	18	(7)	35	(12)	16	(10)	16	(15)	0.14
Chronic kidney disease	15	(6)	31	(10)	13	(8)	6	(6)	0.26

Treatments									
Remdesivir	78	(32)	163	(54)	90	(57)	60	(58)	0.001
Dexamethasone	150	(62)	260	(87)	143	(90)	84	(81)	0.001
Tocilizumab	0	(0)	2	(1)	14	(9)	20	(19)	0.001
MV on admission	131	(54)	249	(83)	116	(73)	92	(88)	0.001
HFNC on admission	75	(31)	41	(14)	38	(24)	4	(4)	0.001

Outcomes									
ICU outcome, death	109	(45)	148	(50)	73	(46)	52	(50)	0.64
Hospital outcome, death	108	(47)	154	(61)	73	(50)	54	(63)	0.003
CRRT need	67	(28)	83	(28)	45	(28)	43	(41)	0.04
MV duration, days, median (IQR)	9	(0–17)	12	(5–26)	13	(4–27)	13	(6–25)	0.001
ICU LOS, days, median (IQR)	13	(7–23)	15	(8–31)	19	(9–36)	17	(9–31)	0.03

All data are expressed as n (%), unless otherwise defined. Abbreviations: N, number of patients; IQR, interquartile range; APACHE, acute physiology and chronic health evaluation; SOFA, sequential organ failure assessment; COPD, chronic obstructive pulmonary disease; MV, mechanical ventilation; HFNC, high flow nasal cannula; ICU, intensive care unit; CRRT, continuous renal replacement therapy; LOS, length of stay.

Table 2 summarizes the laboratory and clinical characteristics of the whole cohort stratified by the four waves. The ICU-LOS was significantly higher in the third compared to the first wave, whereas duration of mechanical ventilation was lower in the first compared with the other three waves. The first wave had a lower APACHE II score compared to that of the second wave, as well as a lower SOFA score compared with that of the second and fourth waves. The fourth wave had significantly lower albumin and fibrinogen, as well as higher troponin and d dimers, compared with at least one of the other three waves.

**Table 2.** Laboratory findings and respiratory parameters on ICU admission

**Total n=805**	**1st wave n=243**		**2nd wave n=300**		**3rd wave n=158**		**4th wave n=104**		**P value**
Laboratory tests									
WBC count, 109/L	9.6	(6.4–13.6)	10.3	(7.1–14.2)	11.8	(6.9–15.4)	11.6	(7.2–16.4)	0.04
Neutrophil count, 109/L	8.2	(5.1–12.1)	8.7	(6.0–12.6)	10.1	(5.8–13.7)	10.1	(6.3–14.7)	0.04
Lymphocyte count, 109/L	.80	(.55 –1.17)	.78	(.50–1.10)	.67	(.49–0.94)	.76	(.49–1.16)	0.08
NLR	9.7	(5.5–15.7)	12.2	(6.9–19.6)	13.1	(8.0–21.5)	13.4	(7.9–25)	0.001
Hemoglobin, g/dL	12.6	(11.4–13.7)	12.1	(10.3–13.7)	12.5	(10.9–13.7)	12.0	(10.3–13.4)	0.12
Platelet count, 109/L	236	(182–306)	247	(187–317)	239	(174–309)	239	(169–301)	0.71
Fibrinogen, mg/dL	600	(497–714	582	(485–701)	551	(481–679)	551	(385–687)	0.009
D-dimers, mg/L	1.3	(.7–2.6)	1.6	(.9–4.3)	1.5	(.8–3.4)	2.2	(1.0–7.2)	0.003
Ferritin, ng/mL	718	(278–1710)	628	(318–1941)	602	(286–1941)	491	(189–1328)	0.16
Albumin, g/dL	3.3	(2.9–3.6)	3.1	(2.9–3.5)	3.2	(2.8–3.6)	3.1	(2.7–3.5)	0.007
Sodium, mEq/L	139	(136–142)	140	(137–144)	140	(138–144)	141	(138–145)	0.001
Creatinine, mg/dL	.9	(.7–1.2)	.9	(.7–1.3)	.8	(.6–1.3)	.9	(.7–1.3)	0.80
AST, IU/L	44	(26–70)	37	(26–68)	35	(23–62)	35	(21–54)	0.08
ALT, IU/L	36	(21–54)	32	(19–56)	26	(16–500	30	(17–61)	0.09
LDH, IU/L	438	(333–610)	474	(357–643)	458	(348–629)	463	(302–591)	0.29
hs-cTnI, ng/L	18	(8–61)	24	(11–79)	24	(8–62)	39	(18–103)	0.001
Procalcitonin, ng/mL	.30	(.12–1.17)	.24	(.11–0.63)	.23	(.10–0.55)	.20	(.10–0.54)	0.19
CRP, mg/dL	12	(5–19)	11	(6–17)	9	(4–17)	9	(3–16)	0.04

Respiratory parameters									
pH	7.39	(7.30–7.45)	7.33	(7.26–7.40)	7.34	(7.26–7.41)	7.29	(7.24–7.36)	0.001
PaCO2, mmHg	40	(33–47)	43	(35–51)	41	(36–47)	43	(37–52)	0.005
Bicarbonate, mEq/L	23	(20–26)	22	(19–24)	22	(20–24)	21	(19–24)	0.007
Lactate, mmol/L	1.6	(1.2–2.1)	1.6	(1.3–2.2)	1.6	(1.2–2.3)	1.8	(1.4–2.5)	0.04
PaO2/FiO2	123	(89–171)	114	(84–181)	137	(90–180)	138	(94–213)	0.17
Respiratory rate, breaths/min	24	(22–27)	25	(21–28)	25	(23–28)	25	(22–28)	0.22
Tidal volume, mL	480	(450–500)	460	(420–500)	450	(400–480)	450	(400–480)	0.001
PEEP, cm H2O	12	(10–15)	12	(10–15)	13	(10–15)	13	(10–16)	0.22
Plateau pressure, cm H2O	26	(24–29)	26	(24–28)	26	(23–28)	27	(24–28)	0.62
Driving pressure, cm H2O	13	(12–15)	12	(11–14)	12	(10–14)	12	(11–14)	0.10

Data are expressed as median (IQR). Abbreviations: ICU, intensive care unit; N, number of patients; WBC, white blood cell; AST, aspartate aminotransferase; ALT, alanine aminotransferase; LDH, lactate dehydrogenase; hs-cTnI, high sensitivity cardiac troponin I; CRP, C-reactive protein; PEEP, positive end expiratory pressure.

### Survival analysis

**Fig. 2.**
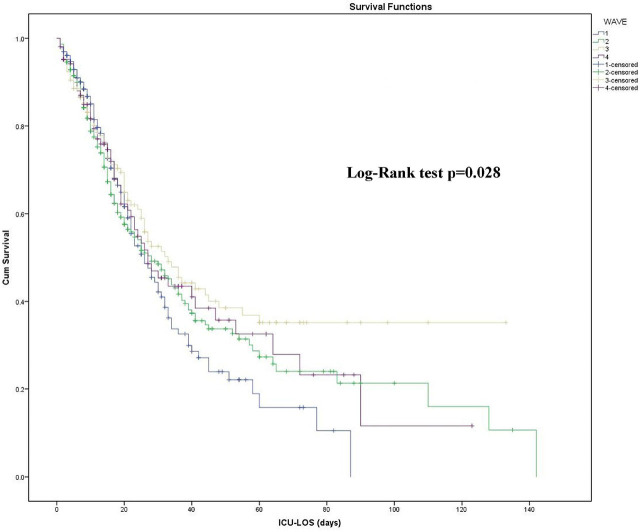
Kaplan-Meier survival curves among the four waves. Abbreviations: ICU-LOS, intensive care unit-length of stay.

Kaplan-Meier survival curves did not reveal significant differences in ICU mortality among the four waves (Log-Rank test p=0.28), Figure 2. Univariate and multivariate Cox regression proportional hazards analyses did not reveal any of the four waves as an independent risk factor for ICU mortality. On the other hand, age, malignancy, chronic pulmonary disease and SOFA score on admission, were independent risk factors for ICU mortality in the multivariate model, Table 3.

**Table 3.** Univariate and multivariate Cox proportional hazards models

	**B**	**P-value**	**Odds Ratio**	**95% CI**	

				**Lower**	**Upper**
Univariate model					
Wave 1st (reference)					
Wave 2nd	−.067	.602	.935	.725	1.205
Wave 3rd	−.287	.062	.750	.555	1.015
Wave 4th	−.135	.430	.874	.625	1.221
Multivariate model					
Sex	.070	.588	1.072	.833	1.381
SOFA score	.145	.000	1.157	1.106	1.209
Wave 1st (reference)					
Wave 2nd	−.150	.297	.861	.650	1.140
Wave 3rd	−.208	.227	.812	.580	1.138
Wave 4th	−.160	.424	.852	.576	1.261
Hypertension	−.085	.467	.918	.729	1.156
Diabetes	−.153	.248	.858	.662	1.112
Obesity	.001	.996	1.001	.701	1.430
Cardiovascular disease	.244	.062	1.277	.988	1.651
COPD	.462	.002	1.588	1.178	2.141
Malignancy	.424	.010	1.529	1.109	2.107
Chronic kidney disease	.112	.568	1.118	.762	1.642
Remdesivir	−.215	.094	.807	.627	1.037
Dexamethasone	.122	.449	1.129	.824	1.547
Tocilizumab	−.734	.052	.480	.229	1.007
Age quartile 1st (reference)					
Age quartile 2nd	.113	.596	1.119	.738	1.698
Age quartile 3rd	.608	.002	1.838	1.239	2.724
Age quartile 4th	.849	.000	2.337	1.580	3.455

Abbreviations: CI, confidence interval; SOFA, sequential organ failure assessment; COPD, chronic obstructive pulmonary disease.

### Sensitivity analysis

**Fig. 3.**
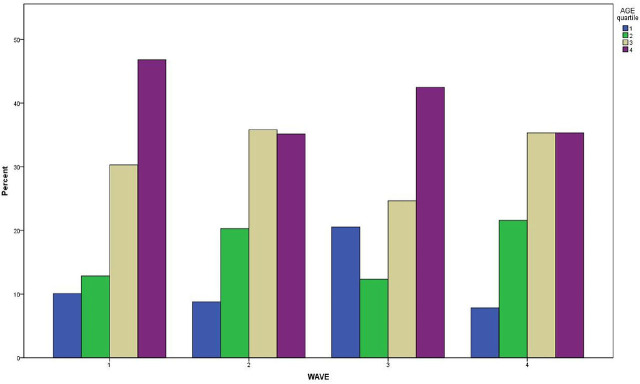
Distribution of age quartiles in non-survivors among the four waves.

**Fig. 4.**
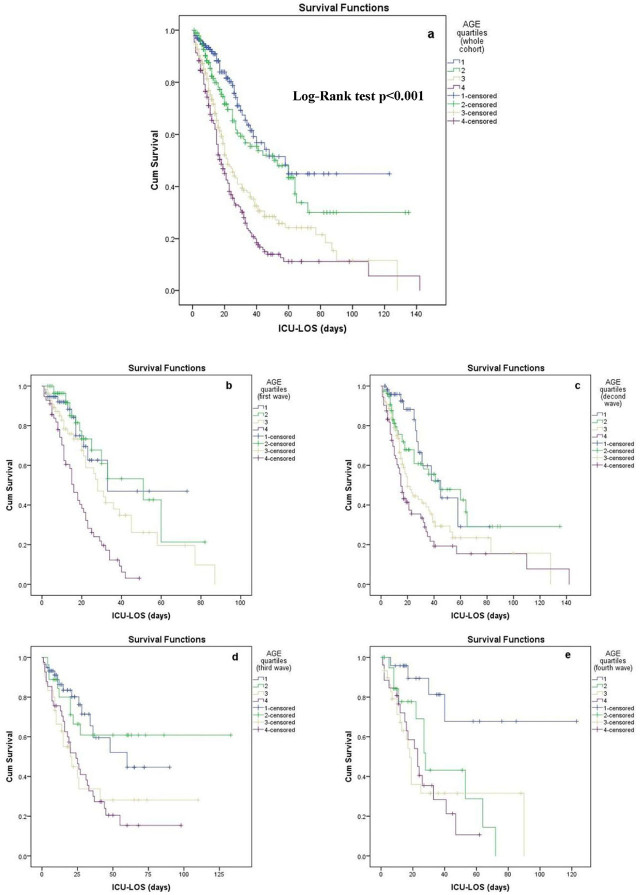
Kaplan-Meier survival curves of age quartiles a) in the overall cohort, b) in the first wave, c) in the second wave, d) in the third wave, and e) in the fourth wave. Abbreviations: ICU-LOS, intensive care unit-length of stay.

We performed a prespecified secondary analysis to explore the association of age with ICU mortality in each pandemic wave. Figure 3 depicts the distribution of age quartiles in non-survivors among the four waves (chi-squared test p=0.08). Figure 4 (a), which demonstrates the Kaplan-Meier survival curves of age quartiles in the overall cohort, showed significant differences in ICU mortality among them (Log-Rank test p<0.001). Figure 4 (b–e) illustrates Kaplan-Meier survival curves of age quartiles for each pandemic wave, and Table 4 summarizes the results of pairwise Log-Rank tests for each wave separately. The fourth age quartile (patients over 76 years old) had significantly lower survival rate compared to both the first and second ones in the first three waves. It also had lower survival rate compared to the first quartile in all four waves, as shown in Table 4. Finally, the 3^rd^ and 4^th^ age quartiles were independent risk factors for ICU mortality in the multivariate model, Table 3.

**Table 4.** Log Rank pairwise comparisons between the age quartiles for each pandemic wave

	**Age quartiles**	**1**		**2**		**3**		**4**	
		**Chi-Square**	**P value**	**Chi-Square**	**P value**	**Chi-Square**	**P value**	**Chi-Square**	**P value**
Age quartiles				first wave					
	1			0.062	0.803	1.732	0.188	14.845	0.000
	2	0.062	0.803			2.727	0.099	22.948	0.000
	3	1.732	0.188	2.727	0.099			11.988	0.001
	4	14.845	0.000	22.948	0.000	11.988	0.001		

				second wave					
	1			0.785	0.376	7.082	0.008	15.125	0.000
	2	0.785	0.376			4.591	0.032	12.535	0.000
	3	7.082	0.008	4.591	0.032			2.530	0.112
	4	15.125	0.000	12.535	0.000	2.530	0.112		

				third wave					
	1			0.005	0.941	7.056	0.008	9.876	0.002
	2	0.005	0.941			4.955	0.026	7.594	0.006
	3	7.056	0.008	4.955	0.026			0.03	0.863
	4	9.876	0.002	7.594	0.006	0.03	0.863		

				fourth wave					
	1			7.484	0.006	10.813	0.001	12.182	0.000
	2	7.484	0.006			0.459	0.498	1.944	0.163
	3	10.813	0.001	0.459	0.498			0.001	0.969
	4	12.182	0.000	1.944	0.163	0.001	0.969		

## Discussion

This study describes the characteristics and the clinical outcomes of all patients who were hospitalized in a large ICU in Athens, Greece, with severe COVID-19 pneumonia, throughout the whole period of the pandemic, according to the four pandemic waves as they were defined by the predominance of different SARSCoV-2 variants. The main findings of the present study are as follows: there were not significant differences in ICU mortality rate among the four pandemic waves. Age, malignancy, chronic pulmonary disease and SOFA score were independent predictors of ICU mortality, but none of the pandemic waves was an independent risk factor. Specifically, age significantly influenced ICU mortality across all waves, with patients of the fourth age quartile (i.e., over 76 years) showing the lowest survival rate.

Data evaluating differences in mortality of COVID-19 ICU patients across the entire pandemic period are scarce [ 21, 22, 23, 24]. Although multiple studies focusing exclusively on critically ill patients have provided comparisons for mortality between consecutive waves, they are usually limited in the first year of the pandemic [13, 14, 18, 19]. Strikingly, the majority of the studies in patients with COVID-19 requiring critical care did not show significant improvement in mortality throughout the consecutive pandemic waves [20, 24]; in some of them, even an increase was observed temporarily [15, 21,22,23]. This is in contrast with other reports including patients with COVID-19 hospitalized in general wards, showing a decline in mortality after the initial surge of the pandemic [33], mainly attributed to effective treatments, including antivirals and corticosteroids along with vaccination programs.

In a Danish database among patients admitted to the COVID ICU, the mortality rate was similar during and after the first wave, whereas higher age and the burden of comorbidities were identified as risk factors for fatal outcome [20], in agreement with our results. Moreover, in a national cohort study involving all critically ill COVID-19 patients, hospitalized in French ICUs up to July 2021, an increase of hospital mortality during the period from January through June 2021 compared with the first surge was reported, whereas a decreasing trend of mechanical ventilation, vasoactive agents, and CRRT need was observed [21]. This is in agreement with the finding of the present study also showing an in-hospital mortality significantly higher during the same time, amid the dominance of the Alpha variant in our country.

In Australia, the in-hospital mortality rate for COVID-19 ICU patients was higher during the third wave (associated with the Delta variant) compared to the first wave. This increase was especially pronounced among patients who required mechanical ventilation, even though the APACHE II scores at admission were similar across all three waves [22]. On the other hand, in the Netherlands, mortality rates among critically ill COVID-19 patients varied over time, with higher mortality observed during the initial surge of each of the three waves. This increase may have been driven by the heightened virulence of the circulating virus strain and the population’s lack of immunity against it at the time [23]. A multicenter cohort study conducted in Spain, Andorra, and Ireland also reported consistently high mortality rates among critically ill COVID-19 patients [15]. The study compared the second and third waves (July 2020 to March 2021) with the first wave (February to June 2020) and found no significant difference in adjusted ICU mortality rates between the waves. However, higher mortality rates were observed during the initial surge phases of the second and third waves.

The above-mentioned studies showing no improvement in mortality confirm earlier reports demonstrating fewer intubations but higher [14, 18] or unchanged [19] mortality among patients with COVID-19 requiring invasive mechanical ventilation after the initial surge, despite the significant treatment modifications including antivirals, and steroids [ 7,8,9,10, 13]. In a meta-analysis including patients from 43 countries, Xourgia et al [34] found that mortality of intubated patients with COVID-19 had not improved since the start of the pandemic until July 2021. Similarly, in a recent analysis including 25 studies [35], no significant difference in in-hospital or ICU mortality was observed when the first wave was compared with the subsequent ones.

Only a few studies described improvements in critical care outcomes, including decreased mortality, following the initial surge in early 2020 [13, 14, 36, 37]. Indicatively, a large observational study evaluating patients admitted to a single ICU in Bronx, New York, exhibited a steady decrease in mortality rates, during the second and third COVID-19 waves (mortality decreased significantly from the first to the third wave (57% vs 37%, respectively) [37]. The reductions in mortality were linked to several factors, such as increased familiarity with the novel coronavirus and the accessibility of proven treatments, including antivirals and steroids. However, a reversal in the initial drop of mortality during the following waves in certain care centers has been reported [14, 38].

The question that emerges from both the present and previous studies, conducted in different countries, is about the reasons for the lack of improvement in terms of ICU mortality across the consecutive waves of the COVID-19 pandemic. Theoretically, the progress in clinical experience along with the advent of new treatments, including steroids and antivirals, could result in an improved clinical outcome. Despite these substantial changes, the results are inconsistent. Although no clear explanation can be given, the fact that, despite the lack of available effective treatments during the first wave compared to the subsequent ones, its ICU mortality was not higher, highlights the great importance of ICU supportive care and management even in settings of limited resources and increased burden, such as the initial outbreak of a pandemic.

According to the results of the present study, both illness severity scores (APACHE II, SOFA) were different among the four pandemic waves. Moreover, SOFA score was an independent risk factor for ICU mortality, according to the results of the multivariate Cox regression analysis. However, age and the various comorbidities were not different among the four waves. Specifically, age significantly influenced ICU mortality across all waves. Taking the aforementioned findings into consideration, we could say that although the clinical severity among ICU patients differed significantly, ICU mortality was not different among the four waves. A plausible explanation for this could be that age [26], and comorbidities [39], have superior impact on COVID-19-related ICU mortality compared to clinical severity, which could largely explain the similarity in ICU mortality across the four periods.

Given that age affects the clinical outcome of COVID-19 [25, 26], we categorized the study population into age quartiles. A pre-planned secondary analysis was then conducted to assess the relationship between age and ICU mortality across each pandemic wave. Similar to other studies [15, 20], we observed that mortality remained consistently high and was strongly linked to older age. Notably, our ICU did not face significant strain during the pandemic, as a substantial number of new ICU beds were made available. This allowed admissions to proceed without restrictions based on age or comorbidities. Consequently, the patient population in this study reflects an unselected, real-world cohort.

Certain limitations of the present study should be acknowledged. First, as a single-center study, the findings reflect a certain geographic area. However, in contrast with the multicenter studies, this design ensures homogeneity, since similar criteria of ICU admission as well as of clinical management were followed, allowing comparisons among the waves. Therefore, this limitation could be rather considered a strength. Second, SARS-CoV-2 genotyping was not routinely performed at the time of the pandemic, but it was instead inferred from the time frame during which each variant was dominant in our country. Third, we did not provide data regarding patients admitted during the same periods across all departments served by our ICU, in order to interpret the results more comprehensively.

Our study covered a longer period (March 2020–April 2022), and more SARS-CoV-2 variants compared to the majority of similar studies exploring COVID-19 pandemic waves. Since there is no universally accepted time frame defining the pandemic waves, many studies divided time according to “surges” instead of waves. We followed the Greek National Public Health Organization for categorization of the four distinctive pandemic waves in our country, based upon the emergence of different SARS-CoV-2 variants [5, 6].

## Conclusion

In this cohort of 805 critically ill patients with COVID-19 admitted to our ICU, we found no statistically significant change in ICU mortality throughout the pandemic. However, increasing age per se was an independent risk factor for excess mortality. The effect of COVID-19 wave (and consequently of the SARS- CoV-2 variant) on ICU mortality seems to be trivial, and therefore our focus should be shifted to other risk factors, such as age, comorbidities, vaccination, antiviral treatment and adequate ICU resources. These findings along with those of other studies could be useful for modelling the evolution of future outbreaks.
